# The effect of a family-based mindfulness intervention on children with attention deficit and hyperactivity symptoms and their parents: design and rationale for a randomized, controlled clinical trial (Study protocol)

**DOI:** 10.1186/s12888-016-0773-1

**Published:** 2016-03-15

**Authors:** Herman H. M. Lo, Samuel Y. S. Wong, Janet Y. H. Wong, Simpson W. L. Wong, Jerf W. K. Yeung

**Affiliations:** Department of Applied Social Sciences, City University of Hong Kong, Tat Chee Avenue, Kowloon, Hong Kong, SAR China; Division of Family Medicine and Primary Health Care, School of Public Health and Health Care, The Chinese University of Hong Kong, Hong Kong, SAR China; School of Nursing, The University of Hong Kong, Hong Kong, SAR China; Department of Psychological Studies, The Hong Kong Institute of Education, Hong Kong, SAR China

**Keywords:** Attention deficit hyperactivity disorder, Executive functioning, Child development, Mindfulness-based intervention, Family-based intervention

## Abstract

**Background:**

About 4 % of children in Hong Kong have attention deficit hyperactivity disorder (ADHD). The parents of children with ADHD report higher levels of stress and show more negative parenting behavior. Medication and behavior training are evidence-based treatments, but both show significant limitations. In short, medical treatment is not suitable for preschool children and would suppress growth, whereas parents under stress may not be capable of consistently applying behavior management skills. Mindfulness training can improve attention and facilitate cognitive development and overall functioning. It has been widely adopted as a treatment option in health care, but its application in a family context is limited. In this context, a family-based mindfulness intervention (FBMI) has been developed to promote the attention and mental health of children with attention symptoms and to reduce the stress experienced by their parents. This article describes the design and conduct of the trial.

**Methods/design:**

A multicenter, 8-week, waitlist, randomized controlled trial of FBMI is currently being conducted in Hong Kong (from mid-2015 to mid-2016). Its effectiveness will be examined by comparing the participants who receive treatment to those in a waitlist control group. The study population consists of one hundred twenty children with ADHD, or with symptoms of inattention and hyperactivity, between 5 and 7 years of age and their parents. To be included in the study, the children are required to meet or exceed the borderline cutoff score of the Chinese version of the Strengths and Weaknesses of ADHD Symptoms and Normal Behaviors Rating Scale (SWAN-C). The primary outcome measures are the children’s ADHD symptoms and behavior and the parents’ stress. The secondary outcome measures include the children’s overall behavioral problems and performance on the Attention Network Test, the parents’ ADHD symptoms, the parents’ mindful parenting scores, and heart rate variability of parents.

**Discussion:**

This study is probably the first randomized controlled trial of FBMI for young children and their caregivers. A rigorous design and multiple outcome measures are used to examine the effectiveness of FBMI. If the hypotheses are confirmed, FBMI may serve as an additional treatment option for children with ADHD.

**Trial registration:**

This study is registered with the Chinese Clinical Trial Registry (reference number: ChiCTR-IOR-15007292). Registered 28 October 2015.

## Background

Attention-deficit hyperactivity disorder (ADHD) is a childhood psychiatric disorder that is characterized by the core symptoms of inattention, hyperactivity, and impulsivity with an early onset [1]. The prevalence of ADHD is between 1.5 % and 8 %, depending on the diagnostic criteria used [2]. A study in Hong Kong estimated that 3.9 % of adolescents had received a diagnosis of ADHD [3]. More children in the community have reported problems of inattention, and studies have found an association between children with subclinical attention problems and problems with peer relationships and negative parenting [4].

Most children with ADHD have significant impairment of their executive functions (EFs). They show consistently worse performance on EF tasks than their typically developing counterparts [5]. EFs are a set of fundamental mental processes associated with the use of higher cognitive abilities that contribute to overall neuropsychological functioning [6]. The core EFs are cognitive flexibility, inhibition (self-control, self-regulation), working memory, problem-solving, reasoning, and planning [7]. When children exhibit EF deficits, they have greater difficulty moderating their behavior and show problems in working memory recall; self-regulation of mood, motivation, and arousal; internalization of verbal communication; and reconstitution of language and motor behavior [8]. Therefore, EFs are more important than intelligence quotient for school readiness [9]. Children with less self-control (less persistence, more impulsivity, and poorer attention regulation) between 3 and 11 years of age tend to have worse health, earn less, and commit more crimes 30 years later than those with better self-control as children, after controlling for intelligence quotient, gender, social class, and other factors [10].

ADHD is also associated with disturbances in the family and poorer parenting practices [11, 12]. The parents of children with ADHD report less marital satisfaction and more conflict than those of children without ADHD [13]. Challenging child behavior evokes harsh parenting, which is defined by intense hostility and negative emotionality and is hypothesized to influence the development of oppositional and conduct problems via a process of mutual reinforcement [14]. The parents of children with ADHD report higher levels of stress, lower levels of social support and quality of life, and less parenting satisfaction than parents of children without ADHD [15]. In view of the reciprocal and dynamic interactions between children with ADHD and their parents, the treatment of ADHD should consider the promotion of calm and consistent discipline and emotional responsiveness in parenting [16].

Currently, evidence-based treatment for ADHD focuses predominately on the use of medication and behavior therapy, but both options have limitations. Although the use of stimulant medications is generally considered to be evidence-based practice [17], the sustained use of stimulants over time is associated with growth suppression and may induce deleterious side effects that are untenable for many individuals, and thus it should not be preferable for young children [18, 19]. The parents of children with ADHD may learn parenting skills from behavior therapy; however, when they are under stress or have their own psychopathology, they may not be able to properly carry out parenting skills, and may feel frustrated and negatively reinforce parent–child conflicts [20]. A family-based intervention would be advantageous because both children and their parents could benefit from improvement of attention and emotional management.

### Mindfulness and its potential application in children and in a familial context

Mindfulness training has been shown to be an effective tool with which to enhance the self-regulation of attention in individuals with ADHD, in conjunction with other evidence-based treatments [21, 22]. Mindfulness training is expected to improve attention in three aspects: orienting attention, alerting attention, and executive attention [23, 24]. Orienting attention concerns the use of all available sensory inputs in the perceptual field to direct and select situation-appropriate information. Alerting attention refers to sustaining attention by achieving and maintaining a vigilant state. Executive attention involves examining, monitoring, and resolving conflicts among one’s ongoing behavioral reactions to the immediate environment [25]. Mindfulness training may improve the ability to strengthen attentional processes [22, 26]; it not only promotes the EFs of children with ADHD, but also improves parents’ self-regulation in response to their child’s challenging behavior and alters the dysfunctional patterns in their parenting behavior [27].

Evidence of mindfulness-based intervention is emerging, specifically for families with children with ADHD. A randomized controlled trial of mindfulness-based cognitive therapy was conducted in 25 children between 9 and 13 years of age who had a mixture of attention problems and ADHD diagnoses [28]. The children showed significantly fewer attention-related problems after the intervention. A mindfulness-based curriculum developed for 409 children from kindergarten to sixth grade from low-income families and ethnic minorities [29] resulted in improvements in classroom behavior, including attention, self-control, and caring for others. However, no control group was used, and the effects on the children’s other developmental aspects remain unclear. The results of an 8-week mindfulness training course conducted in 22 children between 8 and 12 years of age with ADHD and their parents indicated significant reductions in the children’s ADHD symptoms and in their parents’ inattention and hyperactivity symptoms [30]. In a study of the same program in 10 adolescents aged 11 to 15 with ADHD and their parents, the adolescents, parents, and tutors all reported improvements in attention and behavioral problems [31]. Overall, the results are positive but three of four studies were based on small samples, and except the first study, only a simple pretest-posttest comparison design was adopted for outcome evaluation.

There is increasing evidence for the application of mindfulness-based interventions in Chinese populations [32, 33]. However, this project is one of the earliest attempts to apply mindfulness training in children and families, and to the best of our knowledge, no studies of mindfulness training in parents and young children have been published. With reference to the above overseas studies, mindfulness-based interventions for children with ADHD and their parents may be acceptable and feasible, but the published studies have numerous methodological limitations, including small or heterogeneous samples and uncontrolled or nonrandomized designs. Some studies did not involve parents, and most studies involved children above 8 years old, but not young children.

### Study objectives

The objectives of the project are (1) to reduce the symptoms of inattention and hyperactivity of children with ADHD; (2) to reduce the stress of parents; and (3) to examine the effectiveness of family-based mindfulness intervention (FBMI) in Chinese families of children with ADHD or with symptoms of inattention and hyperactivity. The following hypotheses are proposed.

1. We expect FBMI to reduce the symptoms of inattention and hyperactivity of children relative to those in a wait-list control group.
2. We expect FBMI to reduce the internalizing and externalizing symptoms of children relative to those in a wait-list control group.
3. We expect FBMI to reduce parental stress, achieve better well-being, as reflected in heart rate variability (HRV) parameters, and improve parent mindfulness relative to those in a wait-list control group.

## Methods/design

### Study design overview

The families of children with diagnoses of ADHD or with symptoms of inattention and hyperactivity are being recruited to participate in this study. The rationale for not restricting the study to children with formal diagnoses was that many children are first assessed by professionals after 6 in Hong Kong, and therefore many children with severe inattention and hyperactivity symptoms have not yet received a diagnosis.

**Fig. 1:**
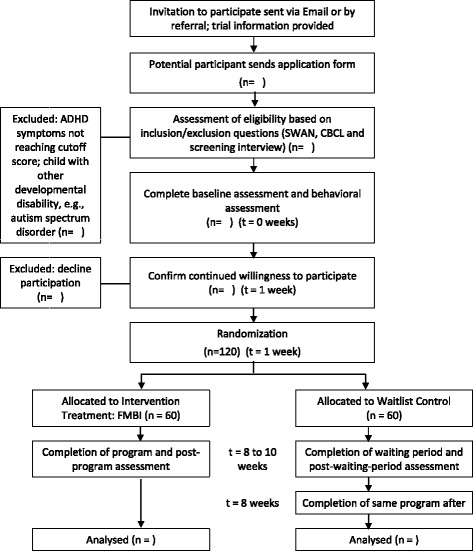
Flow diagram of participant allocation

A flowchart of the recruitment and implementation of this waitlist randomized controlled trial is illustrated in Fig. 1. All eligible families are randomized into a treatment group or a wait-list control group. The FBMI is delivered in a group format. All families in the waitlist control groups will undergo the same program after the posttest of the treatment groups. Three local non-government organizations in Hong Kong are participating in the study. The programs are conducted in the three collaborators’ district family service centers.

The parent program of the FBMI was designed by the Principal Investigator by means of some adaptations to two overseas mindful parenting programs [34, 35]. The program lasts for 6 weeks, and each session lasts 1.5 h. Each parent program can accommodate 15 to 20 parents. A protocol was prepared by the first author, and the session themes and key contents are summarized in Table 1. The parent programs are implemented by instructors employed by the research team or social workers from the three NGOs who have completed training organized by the Principal Investigator. All parent group instructors completed an 8-week mindfulness-based stress reduction program or mindfulness-based cognitive therapy program. Additional training was provided by Dr. Larissa Duncan, the trainer of another mindful parenting program [35], or the first author.

**Table 1:** Child program of Family-based Mindfulness Intervention (developed by Snel, 2014)

Session	Theme	Goal
1	A for attention	- Establish motivation of be attentive and mindful
		- Use breathing as a beginning of exploration of attention
2	Exploring our body	- Introduce mindful movement exercises
		- Expand awareness of body sensation
3	Tasting, Smelling, Hearing, Seeing and Feeling	- Introduce the use of multiple senses in understanding our inner and outside world
4	Feel our feelings	- Learn to be aware and to describe feelings
5	Accepting feelings	- Acknowledge feelings of self and others
		- Experience the importance of accepting feelings
6	Conscious movement	- Bring attention and awareness to self and others
7	The power of awareness and thoughts	- Experience the application of mindful attention and thoughts in daily life
8	Being nice is good	- Consolidate learning
		- Practice of lovingkindness

For the child program, FBMI follows the child mindfulness program “Mindfulness Matters” (the green book for children 5 to 8 years of age) [36]. The program includes four to six children, and each session lasts 1 h. All group instructors possess a professional degree in social work, education, or clinical psychology and have been certified as instructors by completing the 6-day “Mindfulness Matters” professional training program.

During the fourth and sixth sessions of the parent program, 30-min joint activities are included. This design helps the family members to practice mindfulness together and to review their learning and progress in each other’s presence. The session themes of the child program are shown in Table 2. Participants in the wait-list group will receive the same intervention after the families from intervention group complete FBMI. The research team estimated that the study would be conducted in five cycles, with each cycle consisting of 24 families randomized into an intervention group and a waitlist control group.

**Table 2:** Mindfulness training for parents (developed by the HHML)

Session	Theme	Goal
1	Stress of being a parent	- Establish motivation to learn mindfulness for promotion of family health
		- Introduce mindfulness training
		- Introduce body scan
2	Automatic reactions	- Introduce stretching
		- Notice physiological, emotional and cognitive reaction in stressful moments of parenting
		- Use of mindful breathing and nonjudgmental attitude in managing the reaction in parenting
3	Respond to children mindfully	- introduce mindfulness to breath and body
		- Further notice reactive patterns in parenting
		- Introduce three minute breathing as coping
		- Practice deep listening in mindfulness
4	Quality parenting	- Joint session: practice with children, progress review
		- Introduce mindfulness to sounds and thoughts
		- mindful living for ADHD children and family caregivers
5	Facing difficulties with kindness	- Exploring difficulties with mindfulness practice
		- Introduce lovingkindness practice for self-care, and care of others
6	Self-care of parents	- Joint session: practice with children, progress review
		- Care plan of children and self
		- Consolidate learning

All parent and child groups are audio recorded. Among six parent group sessions or eight child group sessions, one session will be randomly selected the teaching integrity and performance. Parent group instructors will be evaluated using Mindfulness-Based Intervention–Teaching Assessment Criteria [37], while child group instructors will be evaluated by a 10 item evaluation constructed by the research team. Independent assessors are recruited by the research team and internal consistency and interrater reliability will be calculated. The child group evaluation form includes two areas, adherence to session protocol, and competence in program delivery. Assessor will give a rating from 1 to 5, in terms of the levels of adherence and competence, in each item of the scale.

**Fig. 2:**
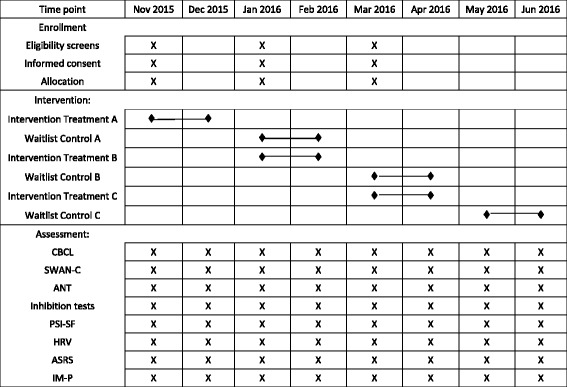
Schedule of enrollment, interventions and assessments

All parents attended a screening interview. The full schedule of enrollment, interventions and assessments is included in Fig. 2. A research team member explained the procedure and research design to them in details. Once the parents agreed to participate in the study with their children, they were invited to sign on the consent forms before the study commenced.

The study protocol has been reviewed by the funder of this study, the Health Care and Promotion Fund from the Food and Health Bureau, The Government of the Hong Kong Special Administrative Region. The study has been given ethical approval from the Research and Contracts Office of the City University of Hong Kong (ref. 3-3-201504_03) and is registered under the Chinese Clinical Trial Registry (ref. ChiCTR-IOR-15007292).

### Study sample

One hundred twenty children between 5 and 7 years of age with ADHD or with symptoms of inattention and hyperactivity and their parents are being recruited to participate in the FBMI. During the screening interview, all outcome measures and demographic information are completed by the parents. The inclusion criteria include (1) children age between 5 and 7 years and (2) a score that meets or exceeds the borderline cutoff of the Strengths and Weaknesses of ADHD Symptoms and Normal Behaviors Rating Scale (SWAN) [38]. The exclusion criteria include (1) children with another developmental disability, such as an intellectual disability or autism spectrum disorder, and (2) the inability of either the parent or the child to attend 80 % of the program sessions.

The randomization procedure is as follows. A 10 × 10 table was created by randomly assigning digits 0 to 9. One row of the table is randomly selected, and the sequence of digits in that row is observed. A participant list is prepared, and the sequence of participants is observed. The first digit will determine the first participant’s group, and so on. Participants with an even digit are assigned to the intervention group, and those with an odd digit are assigned to the control group. After the families are assigned to groups, another research team member contacts the parents by phone, to inform the parent about the results of randomization and to confirm that both the parent and the child will participate in the study. It means that team member who interviews the families is blinded in the assignment process.

## Measurements

### Child functioning

#### Child behavior checklist (CBCL)

The CBCL is used to assess behavioral problems in children by parent’s ratings [39]. It includes 67 items in seven subscales (emotionally reactive, anxious/depressed, somatic complaints, withdrawn, aggressive behavior, attention problems, sleep problems). The first four subscales are classified as internalizing problems, and the following two are classified as externalizing problems, and this factor structure was confirmed in a Mainland China’s study, which reported correlations from 0.38 to 0.71 among the seven subscales and a correlation of 0.75 between internalizing and externalizing problems [40].

#### Strengths and weaknesses of ADHD symptoms and normal behavior rating scales (SWAN)

The scale was originally developed according to the DSM-5 diagnostic criteria for ADHD, and is completed by parents to assess their child’s attention and hyperactivity symptoms [41]. The borderline and cutoff scores were proposed in a local study [38].

#### Child attention network test (ANT)

The test is administered by a research team member and in a computer program [42]. Five fishes are presented in a horizontal row above or below the fixation point. The children are instructed to press a key to indicate in which direction the central fish is pointing and to ignore the flanking fishes. Completion of the task allows the calculation of three scores related to the efficiency of attention networks. Alerting is measured by the additional time required to respond with no cue, compared to the response time to a cue that informs the child that a target will occur shortly. Orienting is measured by the time taken to respond to a cue at the target location minus the reaction time to a central cue. Executive attention is measured as the interference effect of the flanking fish on the child’s score.

### Parent functioning

#### Parenting stress index short form (PSI-SF)

The PSI includes 36 items and was developed to reveal the sources of difficulties and the level of parenting stress [43]. The scale is divided into three subscales: parental distress, parental-child dysfunctional interaction, and difficult child. The Chinese version has been validated [44]. The reliability estimates for the total score and the three subscales were 0.92, 0.86, 0.82, and 0.86, respectively.

#### Parent heart rate variability (HRV)

The HRV is adopted to understand how psychological stress can lead to poor health status, biological data will be used together with psychological measures to evaluate the outcome of the program. Consistent research findings show that psychological distress and negative emotions affect the autonomous nervous system by inhibiting the cardiac parasympathetic system and decreasing HRV [45, 46]. HRV is a measure of cardiac autonomic function in which the cyclic variations in the RR intervals on an electrocardiogram are counted. It is also an early marker of cardiovascular risk [47]. HRV is measured by using ambulatory electrocardiogram to reflect on mother’s autonomous nervous system functioning for three minutes, using Polar heart-rate monitors (Polar Vantage NV, Polar Electro Oy, Finland). HRV is interpreted with the frequency-domain method according to the guidelines for the standard measurement and interpretation of HRV developed by a task force of the European Society of Cardiology and the North American Society of Pacing and Electrophysiology [48]. The indices of frequency-domain analysis include very-low-frequency (VLF), low-frequency (LF), and high-frequency (HF) in absolute values of power (ms2) and normalized units by using Kubios HRV (version 2.2) software.

#### Adult ADHD self-report scale (ASRS)

The ASRS is used to assess parent inattention and hyperactivity symptoms. It includes 18 items, 9 for inattention and 9 for hyperactivity and impulsivity [49]. The Chinese version of this scale has been validated in a sample from Taiwan which showed high intraclass correlations between 0.80 and 0.85, and internal consistency with Cronbach’s alpha of 0.83 to 0.91 [50].

#### Interpersonal mindfulness in parenting (IM-P)

The IM-P scale includes 31 items that assess the parent’s quality of mindfulness specific to his or her family context [51]. The original subscales include listening with full attention, emotional awareness of self and child, self-regulation in the parenting relationship, nonjudgmental acceptance of self and child, and compassion for self and child. The inter-item coefficient ranged from 0.45 to 0.72. The scale validation study in parents with preschool children in Hong Kong has been conducted by the first author (Lo HHM, Yeung JWK, Chan SKC, Ma Y, Siu AFY, Szeto MP, et al. Validating of a Chinese version of the Interpersonal Mindfulness in Parenting (CIMP) questionnaire in Hong Kong and development of a short form. Res Soc Work Pract. In review.).

### Statistical analysis

The baseline characteristics of the intervention group and the waitlist control group will be compared by analysis of covariance for continuous variables and chi-square tests for categorical variables. The baseline factors include the age of the children and the parents, the sex of the children and the parents, the children’s medication status, and the SWAN scores. The effects of FBMI will be tested by analysis of covariance, comparing the FBMI group (arm 1) to the wait-list control group (arm 2). All analyses will be carried out according to the intention-to-treat approach. The participants’ missing values will be imputed using the last-observation-carried-forward method. A two-sided *P* value of 0.05 or less will be considered be statistically significant.

### Estimation of sample size

Because no studies of FBMI have been performed in the families of young children with ADHD, and because the effect sizes of studies of psychosocial treatments vary, the research team adopted an estimated effect size of 0.6 for changes in child attention and behavior. Based on a one-sided type I error of 5 % and 80 % power to detect statistically significant differences between the FBMI and wait-list control groups, the required sample size per arm is 45. Considering an attrition rate of 15 %, more than one hundred six families should be recruited for the study.

## Discussion

Although ADHD is one of the most common mental disorders in early childhood, existing treatments have limitations, and the families of children with ADHD experience high levels of stress that create a great burden to school systems and the community [52, 53]. Poor management of child behavior and family relationships further increase the risks of other comorbid psychopathologic conditions, such as oppositional defiant disorders and conduct disorders in children and major depressive disorders in caregivers [54, 55]. The search for effective treatments to improve the functioning and quality of life of families of children with ADHD should be a priority in the mental health care and education sectors.

We are conducting the first randomized controlled trial of FBMI in Chinese families that contains multiple outcome measures with biomarkers and child attention test. The study includes multiple sites and recruits one hundred twenty families, based on an estimation of the expected effect size. Successful completion of this study and confirmation of the hypotheses will contribute to the evidence base regarding FBMI and treatment options to ADHD. The application of FBMI may also be considered for children with other clinical problems, such as autism spectrum disorder, severe behavioral problems, conduct disorders, depression, and anxiety. Further studies should also consider a 6-month or 1-year follow-up period to verify the sustainability of the treatment effects. Treatment designs that can compare the effects and costs of medication, behavior training, and other methods will be the next step in investigating the efficacy of FBMI.
